# Laparoscopic Resection for Colonic Obstruction Due to Immunoglobulin G4-Related Disease: A Case Report

**DOI:** 10.7759/cureus.78119

**Published:** 2025-01-28

**Authors:** Hitoshi Kameyama, Gen Tomizawa, Toshiyuki Yamazaki, Akira Iwaya, Hideki Hashidate

**Affiliations:** 1 Department of Digestive Surgery, Niigata City General Hospital, Niigata, JPN; 2 Department of Pathology, Niigata City General Hospital, Niigata, JPN

**Keywords:** colon cancer, colonic obstruction, fibrosis, igg4-related disease, laparoscopic surgery

## Abstract

An 89-year-old woman was referred to our hospital with complaints of body weight loss and anemia. Lower gastrointestinal imaging revealed a 3.5-cm stenosis in the descending colon near the splenic flexure. Endoscopy did not pass through the stricture. A biopsy showed no evidence of malignancy or lymphoma. Enhanced abdominopelvic computed tomography showed a thickened descending colon wall with swollen lymph nodes around the colon. Because descending colon cancer was suspected, laparoscopic-assisted left hemicolectomy with D2 lymph node dissection was performed. Hematoxylin and eosin staining showed severe fibrosis and chronic inflammatory cell infiltration. Immunohistochemistry staining showed the number of immunoglobulin G4 (IgG4)-positive cells at 100/high-power field (400×), and the IgG4/IgG ratio was >40%. The serum IgG4 level was measured postoperatively and was within the normal range at 54 mg/dL. The patient was diagnosed as a probable group of IgG4-related disease based on the comprehensive clinical diagnostic criteria for IgG4-related disease.

## Introduction

Immunoglobulin G4-related disease (IgG4-RD) is a multiorgan immune-mediated condition mimicking malignancies, infections, and inflammatory disorders [1]. IgG has four subclasses, numbered IgG1, 2, 3, and 4. IgG4 is the least abundant but is known to be potentially beneficial against allergic diseases. On the other hand, it has also been suggested to be pathogenic to various diseases [2]. IgG4-RD is characterized by IgG4-positive plasma cells and elevated serum IgG4 levels [1]. The 2020 revised comprehensive diagnostic (RCD) criteria for IgG4-RD was proposed by the Research Program for Intractable Disease Ministry of Health, Labor and Welfare Japan, G4 team [3]. The American College of Rheumatology (ACR)/European League Against Rheumatism (EULAR) also published the IgG4-RD criteria [4]. IgG4-RD is a systemic disease, with findings in various sites, including salivary glands, orbit, pancreas and biliary ducts, lungs, kidneys, aorta, retroperitoneum, meninges, and thyroid gland, but rarely in the gastrointestinal tract, particularly the large intestine [5]. In the ACR/EULAR criteria, gastrointestinal lesions are excluded from the entry criteria [4]. In recent years, there have been reports on gastrointestinal lesions of IgG4-RD [4-10]. In particular, colorectal lesions with stenosis are difficult to distinguish from cancer, which influences the decision of treatment strategy and operative procedures. Here, we report a case of laparoscopic surgery for colonic obstruction due to IgG4-RD and review previous reports of colonic lesions due to IgG4-RD.

## Case presentation

An 89-year-old woman was referred to our hospital with complaints of body weight loss and anemia. She lost 4 kg of body weight in one year. Her medical history showed that she underwent subtotal gastrectomy due to a gastric ulcer and appendectomy for appendicitis. She was on medications for hypertension and had no history of allergies. There was no family history of malignant or autoimmune diseases. Physical examination revealed no enlarged lymph nodes or abdominal distention. The patient’s height and body weight were 133.9 cm and 35.1 kg, respectively. Blood tests showed anemia (hemoglobin level: 10.6 g/dL). No inflammation was observed (white blood cell count: 6,640/μL, C-reactive protein level: 0.17 mg/dL). Laboratory data at admission are shown in Table 1.

**Table 1:** Laboratory results CEA: carcinoembryonic antigen; CA 19-9: carbohydrate antigen 19-9

Tests	Reference range	Patient values
White blood cell	3.3-8.6 × 10^3^/μL	6,640
Red blood cell	3.86-4.92 × 10^3^/μL	336
Hemoglobin	11.6-14.8 g/dL	10.6
Hematocrit	35.1%-44.4%	33.4
Platelet	158-348 × 10^3^/μL	322
Blood urea nitrogen	8-20 mg/dL	13.6
Creatinine	0.46-0.79 mg/dL	1.01
Sodium	138-145 mmol/L	138
Potassium	3.6-4.8 mmol/L	5.2
Chloride	101-108 mmol/L	107
C-reactive protein	0-0.14 mg/dL	0.17
CEA	0-5 ng/mL	3.0
CA 19-9	0-37 U/mL	14.7

**Figure 1:**
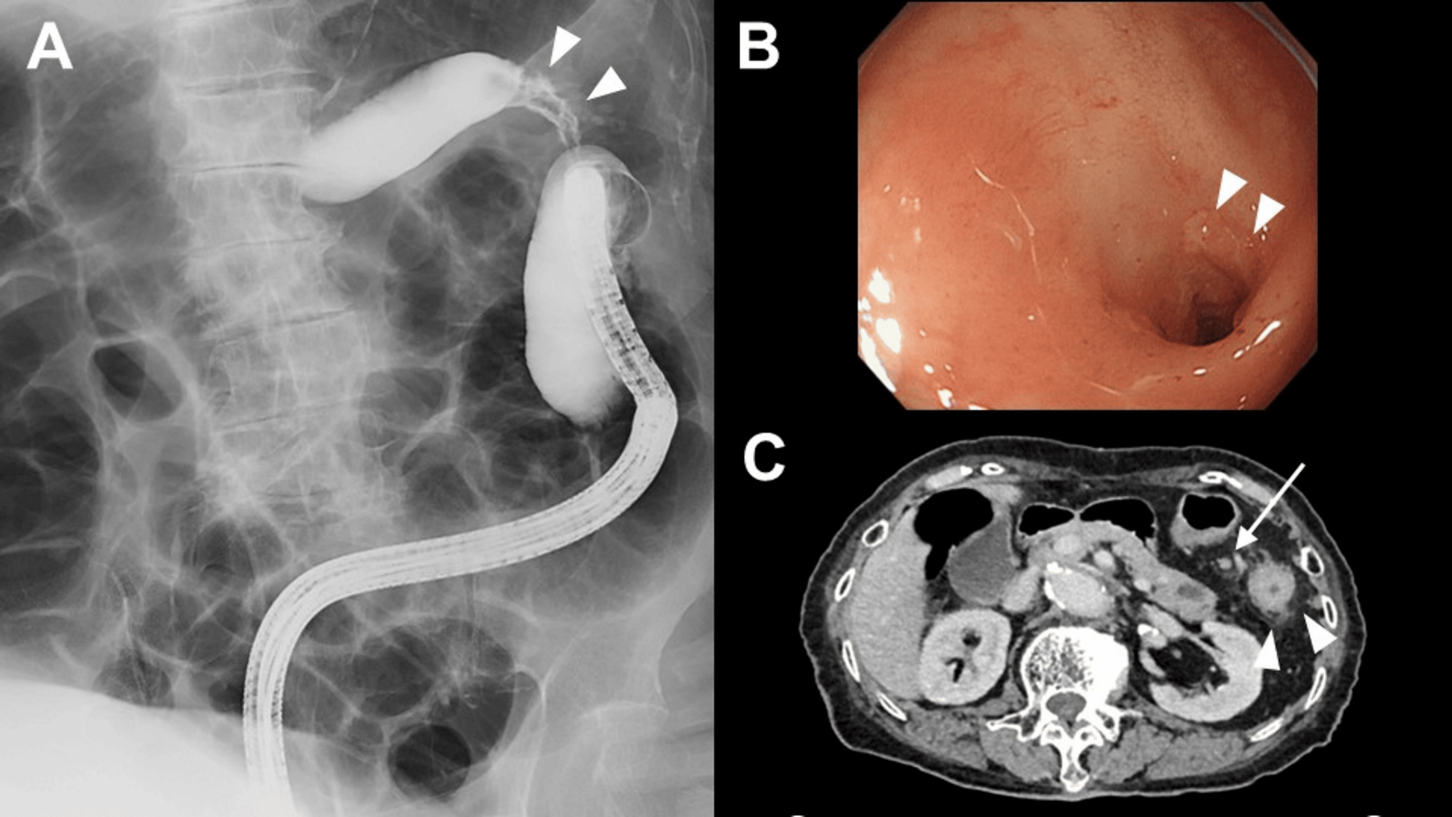
Preoperative findings. (A) Lower gastrointestinal imaging. A circumferential stenosis was observed in the descending colon around the splenic flexure (arrowheads). The length of the stricture was approximately 3.5 cm. (B) Colonoscopy finding. A circumferential stenosis of the descending colon was observed. A longitudinal ulcer was observed in the center of the stenosis (arrowheads). Colonoscopy could not pass through the stenosis. (C) Enhanced abdominopelvic CT. CT scan showed the thickened descending colon wall (arrowheads) and the swollen lymph nodes around the descending colon (arrow) CT: computed tomography

Lower gastrointestinal imaging revealed a 3.5-cm stenosis in the descending colon (Figure 1A).

A colonoscopy revealed a circumferential stenosis of the descending colon near the splenic flexure. An ulcer was observed in the center of the stricture. Endoscopy did not pass through the stricture. A biopsy showed no evidence of malignancy or lymphoma (Figure 1B).

Upper gastrointestinal endoscopy revealed nothing abnormal. Enhanced abdominopelvic computed tomography (CT) revealed a thickened descending colon wall and swollen lymph nodes around the descending colon. CT revealed a cystic lesion in the pancreatic tail, diagnosed as an intraductal papillary mucinous neoplasm, but no evidence of pancreatitis (Figure 1C).

**Figure 2:**
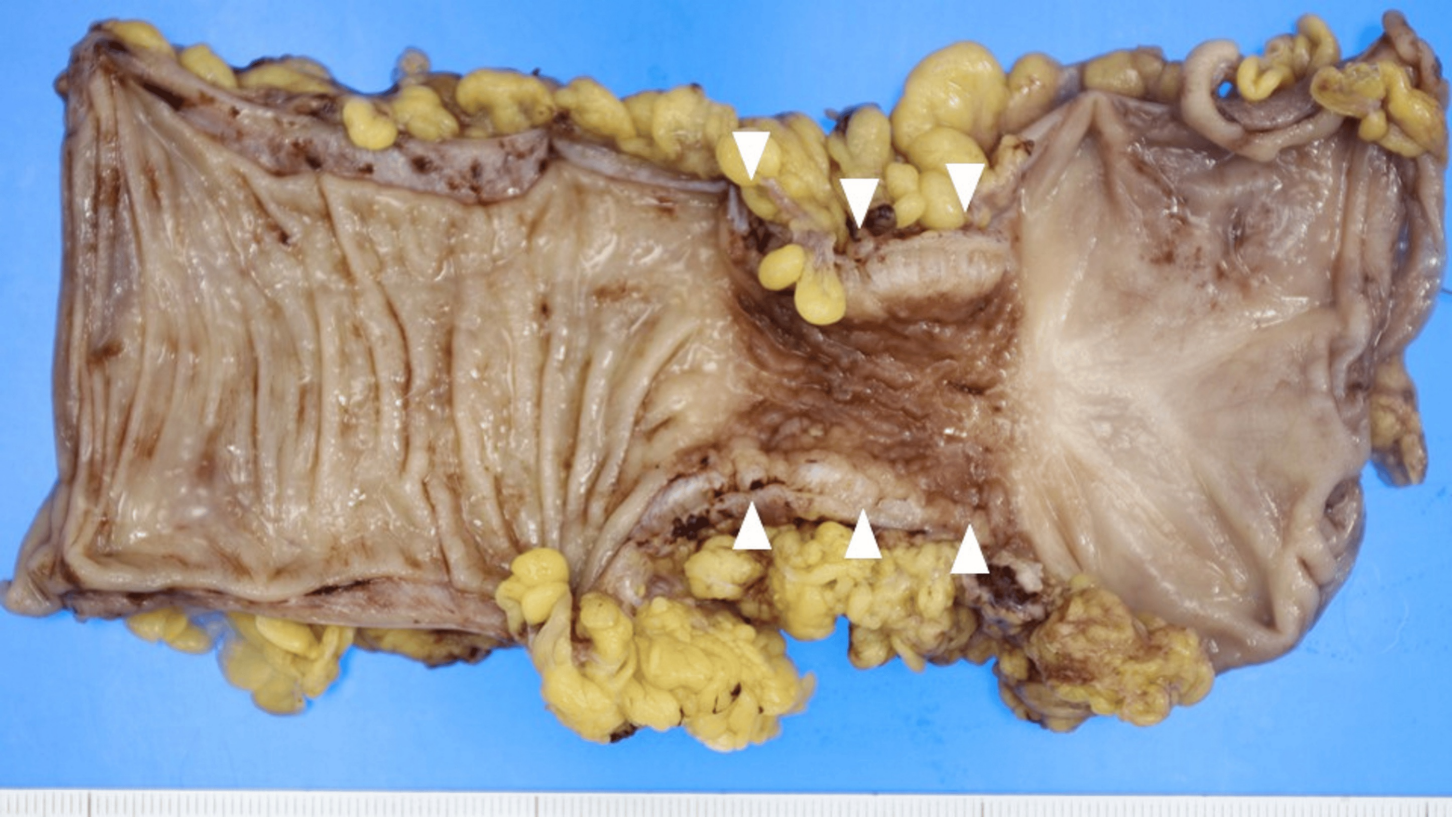
Surgical specimen, showing a 3.5-cm-long circumferential stenosis in the descending colon (arrowheads)

A positron-emission tomography (PET)/CT scan was not performed. Preoperative examination revealed a diagnosis of descending colon stenosis with swollen lymph nodes. Colon cancer, ischemic colitis, and inflammatory bowel disease were considered as differential diagnoses. Laparoscopic-assisted left hemicolectomy was performed using a five-port laparoscopic approach. The omentum covered a stenotic lesion in the descending colon near the splenic flexure. There were no specific inflammatory findings in the abdominal cavity. Assuming colon cancer, the left colonic artery was the main artery. Because descending colon cancer was suspected, D2 lymph node dissection was performed. The operative time was 108 minutes, and the blood loss was 5 mL. The surgical specimen showed a 3.5-cm-long circumferential stenosis (Figure 2).

**Figure 3:**
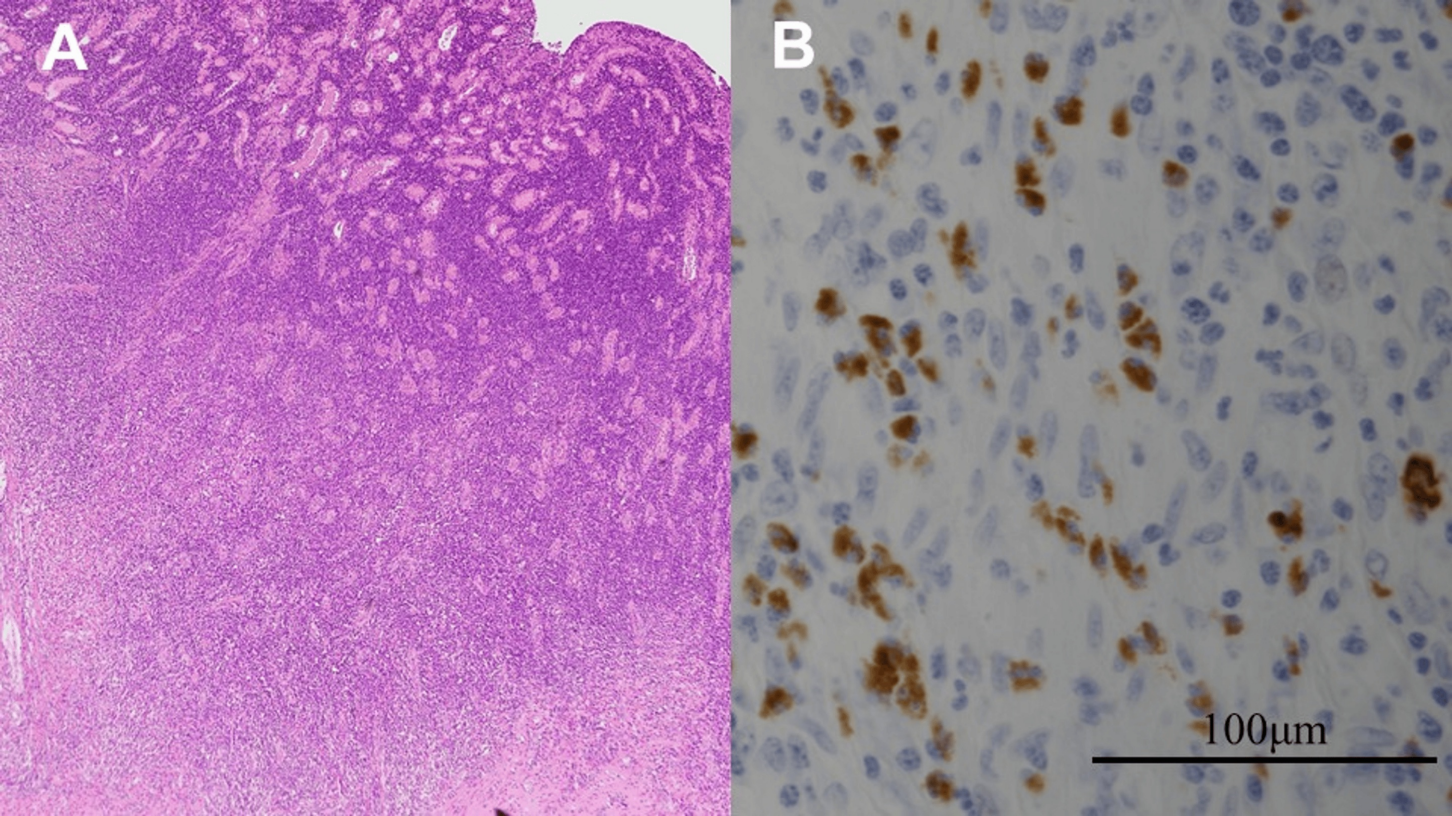
Pathological findings. (A) Hematoxylin and eosin staining showed severe fibrosis and chronic inflammatory cell infiltration. Plasma cells were predominantly infiltrated. There was no evidence of storiform fibrosis or obliterative phlebitis. (B) Immunohistochemistry staining showed more than 100 IgG4-positive cells per high-power field. The IgG4/IgG ratio was >40% IgG4: immunoglobulin G4; IgG: immunoglobulin G

Hematoxylin and eosin staining showed severe fibrosis and chronic inflammatory cell infiltration. Storiform fibrosis or obliterative phlebitis was not evident (Figure 3A).

Immunohistochemistry staining showed the number of IgG4-positive cells at 100/high-power field (400×), and the IgG4/IgG ratio was >40% (Figure 3B). Immunohistochemistry staining was performed using monoclonal IgG antibody (Histofine, Nichirei Biosciences Inc., Tokyo, Japan). The serum IgG4 level was measured postoperatively and was within the normal range at 54 mg/dL.

Postoperative recovery was uneventful, and the patient was discharged six days after the operation. She was followed up for seven months and has shown no IgG4-RD-related symptoms yet.

## Discussion

IgG4-RD can occur systemically in various organs in 58%-88% of patients. IgG4-RD induces many diseases, including Mikulicz’s disease, retroperitoneal fibrosis, and autoimmune pancreatitis. Histopathologic features of IgG4-RD are dense infiltration of lymphocyte/plasma cells and fibrosis [3].

Intestinal lesions of IgG4-RD are rarely reported, especially those that cause colonic obstruction. Only five cases, including ours, have been reported. Mesenteric lesions may cause intestinal stenosis, but cases that cause colonic obstruction are rare [6]. Previous reports have shown colonic obstruction in various sites, including ascending, descending, and sigmoid colons (Table 2). Colonic obstruction due to IgG4 is rare; therefore, its frequency is unknown. However, it could potentially be more common.

**Table 2:** Summary of colonic obstruction due to IgG4-related disease S: sigmoid colon; A: ascending colon; D: descending colon; IgG4: immunoglobulin G4 ^†^Postoperative value

Case	Study	Age (years)	Gender	Preoperative diagnosis	Location	IgG4 value (mg/dL)	Treatment
1	Zhan et al. [5]	50	Male	Inflammation or tumor	S	1,830^†^	Transition from laparoscopic to open sigmoidectomy
2	Hiyoshi et al. [6]	74	Female	Malignant lymphoma	Ileum, A	102^†^	Open right hemicolectomy
3	Matsubayashi et al. [8]	69	Male	Autoimmune pancreatitis	D	1,060	Steroid
4	Syu et al. [9]	40	Male	Cancer suspected	A	169^†^	Open right hemicolectomy
5	Our case	89	Female	Cancer suspected	D	54^†^	Laparoscopic left hemicolectomy

Colorectal stenosis makes differentiation from colorectal cancer difficult. There have been several reports of preoperative diagnosis in cases of small intestinal or colonic lesions [5-10]. Repeated biopsies reportedly help avoid surgery by providing a differential diagnosis, prompting consideration for steroid therapy [9]. However, for intestinal stenosis, a biopsy is also difficult, and PET/CT has also been reported to show a typical peritoneal dissemination pattern of abdominal malignant disease [11]. Preoperative differentiation between IgG4-RD and malignant disease remains difficult.

Because the patient experienced body weight loss and anemia, and the colonoscopy could not pass, minimally invasive laparoscopic surgery was selected. According to the 2020 revised RCD criteria for IgG4-RD, our case met clinical and radiological features (Item 1) and pathological diagnosis (Item 3) and was diagnosed as a probable group. In the present case, serum IgG4 was not measured preoperatively, and the postoperative IgG4 value was within the normal range. The serum IgG4 level value could have decreased or been false negative after resection. In cases where a malignant disease has not been definitively diagnosed, the measurement of serum IgG4 levels may be considered [6]. Pretreatment IgG4 measurement is important for treatment decision-making. In addition, it will help clarify the pathogenesis of IgG4-RD. High serum IgG4 level is neither sensitive nor specific enough for diagnosis [1]. No preoperative diagnosis was made in previous reports, except for one case in which autoimmune pancreatitis was diagnosed (Table 2).

IgG4-RD may also be associated with inflammation of the mesentery and retroperitoneum, and there have been cases in which other organs are involved. In some cases, laparoscopic surgery is difficult. Zhan et al. reported a case where laparoscopic surgery was initiated, but the patient underwent laparotomy because of suspected bladder involvement [5]. In our case, there was no particular inflammation of the mesentery.

## Conclusions

The most important factor in diagnosing IgG4-RD is suspecting it. However, distinguishing IgG4-RD from malignant diseases is difficult. Colorectal lesions in IgG4-RD are rare and remain unreported, so future studies are needed.
